# Changes in Sedimentary Redox Associated with Mussel (*Mytilus edulis* L.) Farms on the West-Coast of Scotland

**DOI:** 10.1371/journal.pone.0045159

**Published:** 2012-09-24

**Authors:** Thomas A. Wilding

**Affiliations:** Department of Ecology, Scottish Association for Marine Science, Scottish Marine Institute, Oban, Argyll, United Kingdom; University of Southampton, United Kingdom

## Abstract

Aquaculture is growing rapidly in response to an increasing demand for protein and the over-exploitation of wild fisheries. Mussel (family Mytilidae) production has doubled over the last decade and currently stands at 1.5 million tonnes production per annum. Mussels produce organic biodeposits which are dispersed around the production site and, potentially, impact the receiving environment in a number of inter-linked ways. The reported benthic impacts that occur, primarily through the accumulation of these biodeposits and associated organic enrichment, vary widely between studies. The objectives of this research were to determine the nature of the relationship between sediment redox (a proxy for oxygenation) and farm-proximity and covariables whilst accounting for, and quantifying, differences in redox between sites. Sediment cores (N = 159) were taken remotely around a random sample of mussel farms, redox was measured at 10 mm sediment depth and linked to farm-distance and sediment organic/shell content and particle size, using an additive, mixed, weighted regression model. Redox varied considerably between sites and there was a highly significant reduction (50 mV) in redox adjacent to the mussel lines. Redox increased non-linearly with distance, rising rapidly at >7 m from the farm edge. The modest reduction in sediment oxygenation in close proximity to mussel farms reported here suggests that farms located over sediments characterised by pre-existing oxygen stress are likely to exacerbate benthic species impoverishment associated with reducing sedimentary conditions whilst those located over highly oxygenated sediments are likely to increase benthic productivity.

## Introduction

Aquaculture is growing rapidly in response to an increasing demand for protein and the over-exploitation of wild fisheries [1]. Sectors of the aquaculture business that are growing include fish (e.g. salmon) but such species are reliant on protein- and oil-based feeds that are derived from increasingly limited marine and/or terrestrial sources [2], [3]. Unlike fin-fish culture bivalve culture does not require feed inputs from the grower [4] and has the additional advantages of relieving pressure on wild shell-fish fisheries [5], potentially replacing historically depleted shell-fish stocks [6] and being amenable to small-scale, artisanal communities [7]. Mussel production, which has doubled in the last decade, currently exceeds 1.5 million tonnes per annum (FAO Statistics).

Mussels are farmed by stocking suitable sites with juveniles and allowing them to grow for a period prior to harvest. Sites can consist of areas of seabed (reviewed in [6]) or the water column. Mussels can be supported in the water column by bamboo poles (notably in Thailand) [8], other wooden poles (e.g. ‘bouchots’ in France) [9], underneath rafts as in the Spanish rias [10]–[12] or, as reported here, suspended on lines strung between floats. Mussels feed by pumping water through specially adapted gills that act as filters and trap particulate material [13]. Trapped particles are wrapped in mucus and either ingested or ejected as pseudofaeces. True faeces and uningested pseudofaeces (collectively known as biodeposits) are dispersed within and around the farm according to currents and water depth and, to some extent, accumulate on the seabed [13].

Mussel culture impacts can be divided into two broad inter-related categories, benthic and water column. Impacts on the water column occur on the scale of the supporting water body and often relate to changes in seston [14]–[16] or nutrient cycling (both of which may involve the benthic habitat) [13]. Benthic impacts, the focus of this study, are initiated by the accumulation of biodeposits on the seabed. Under the Pearson and Rosenberg [17] paradigm this organic enrichment increases sediment oxygen demand which, if not met from the overlying water column, results in a series of changes in the benthos [18]: the sedimentary redoxcline (below which anaerobic respiration results in the reduction of sulphates to sulphides) moves upwards (e.g. [19]) with the concomitant extirpation of larger, longer-lived burrowing fauna and, ultimately, the exclusion of all but a low-diversity sulphide tolerant assemblage. Mussels are generally cultured in shallow, non-dispersive coastal sites [20], that are associated with moderate water flow but which are relatively unexposed [21]. Given that mussel biodeposits sink [22] their accumulation in and around mussel farms, and the subsequent elevated oxygen demand, is inevitable (although not necessarily easily detected) and is likely to be influenced by site-specific factors such as depth and current exposure [23].

The organic enrichment of sediments that has been recorded to occur under some mussel farms can be monitored in several ways including the assessment of mass loss-on-ignition (LOI). LOI at 250°C is a measure of the loss of labile organic carbon (commonly associated with the greatest oxygen demand) whilst LOI at higher temperatures (e.g. 500°C) is a measure of the total organic content (labile and refractory material) [24]. Measurement of the reduction/oxygenation status of sediments has routinely been conducted in the aquaculture industry using ‘redox’ probes. Redox probes measure the electrical potential between a platinum electrode and a saturated solution of silver chloride [25]. When expressed on the hydrogen scale (and corrected for temperature) redox measurements of <0 mV, classified as polluted in relation to organic enrichment by Wildish et al [26], indicate that the sediment is likely to contain sulphide metabolites toxic to most macrobenthic species [18]. Whilst measuring sulphide concentration directly is possible it necessitates laboratory analysis and, therefore, incurs a considerable time (and cost) penalty when compared with the use of redox probes which can be taken into the field.

Whilst a reduction in redox around fish-farms is routinely observed [27] there are more contradictory reports of significant declines in redox potential associated with mussel farms (based on comparing near- and farm-distant stations) even in the same location (e.g. Prince Edward Island, Canada, compare Miron et al [28] and later work by Hargrave et al [29]). In addition to biodeposits, living shells are inevitably lost to the seabed from mussel farms (e.g. via storm damage, bird predation and/or routine farm maintenance) and these are rapidly predated leaving empty shells [30]. Collectively, empty shells (in various states of decomposition) is termed shell-hash and such material may influence the accumulation of biodeposits by increasing the benthic-boundary layer thickness (hash is a rough surface when compared with mud) [23].

The absence of detectable impacts around mussel farms is often attributed to sufficiently dispersive conditions (e.g. [31], [32], [33]). Dispersive conditions limit the accumulation of biodeposits and, together with the high water exchange, make it unlikely that a reasonably sized sampling programme would demonstrate farm-related changes above background variability. In such circumstances testing hypotheses of no difference between near-and farm-distant stations is not particularly revealing, it merely tests the power of whatever sampling programme is being used [34]. However, there is currently very little information on the nature of the functional relationship between distance from mussel farm (and covariables) and redox or variability between sites [23]. Understanding this parameter will inform regulators and developers on the likely spatial extent (i.e. the footprint size) and degree of change associated with mussel farms.

The objectives of this research were twofold: (i) to establish the relationship between redox and distance from mussel lines to inform managers about the likely footprint size of mussel farms and (ii) to establish the extent to which redox variability differed between mussel farm sites and as a function of organic enrichment (the main hypothesised driver of mussel-farm related changes), sediment particle size (closely aligned with current speed) and shell content (potentially a factor enhancing organic particle entrapment). These objectives were achieved and the functional relationship between distance from mussel lines and redox is described.

## Methods

### Site selection

In Scotland, a single mussel farm can comprise of one or more sites, each consisting of floats or rafts from which mussels are suspended on ‘droppers’ that hang into the water column. The lines are commonly in multiples of 220 m (a standard rope length), are supported by numerous equidistantly spaced floats and are normally deployed in an array running parallel to the shore or across bays (with typically 5–15 m separation between lines). In order to examine changes in the benthic environment around mussel farms, sites that were within 50 km of the laboratory were selected (for logistical reasons) where the outer line hosted a standing crop of mussels (mussels of harvestable age i.e. >two years old) that was adjacent to an area of sediment (as opposed to bedrock) in which sampling could occur. Inference to all farms within the sampled population was desired and, therefore, mixed modelling was the appropriate statistical framework for data analysis [35]. Assigning ‘Site’ as a random factor meant that direct comparisons between lochs, or sites within lochs, were not appropriate but that all site-specific factors not accounted for in the measured covariables would be accounted for (but not distinguished) in the random term [35]. Permissions were obtained from the farm owners/managers prior to sampling.

### Sample collection

In order to achieve the objectives sediment sampling only occurred at pseudo-random distances (see below) to the mussel lines, or sections of line, that hosted a mussel crop. The presence of the crop was clearly indicated by the height of the line-float in the water and was corroborated by slightly raising one or more droppers. Samples were not taken from within the farm site. Sediment samples were collected using a Craib corer [36]. Random positioning of the boat around the farm was initially attempted. However, the time taken to locate the random station (identified using GIS) and the frequent failure of the coring device to take cores in shelly sediments necessitated a different approach. Cores were, therefore, collected on a pseudo-random basis where the actual position of sampling was determined by the drift of the boat (current/wind dependent) and success or otherwise of the coring attempt.

The location of each core (total N = 159) was recorded by noting the position of the A frame (via a dedicated A-frame mounted dGPS aerial) from which the corer was lowered vertically to the seabed. The position was noted as soon as the corer reached the bottom (as indicated by a slackening of the winch wire), the survey vessel regaining its position (if necessary) prior to raising the corer.

The distance of each sample to the respective mussel line (factor ‘Distance’) was determined using GIS. However, samples with calculated distances of <1 m were recoded as 1 m distant to reflect the estimated resolution of the sampling system. Each core was processed in the following order (detailed below): redox was measured at 10 mm sediment depth, the top 10 mm of sediment was removed and subsamples were then subject to analysis by mass-loss on ignition and acid digestion and particle size analysis. Sampling occurred between July and September 2010.

### Measuring redox and sediment sample collection

Redox was measured immediately following collection using a redox probe (Russel pH Ltd, Auchtermuchty, Scotland, Model CMPtr 106/300 mm). Prior to use the probe was checked against a standard solution [25] and the sediment-water interface temperature recorded. The probe was inserted 10 mm into the sediment and the redox value recorded once the reading had stabilised (generally after two to three minutes). Estimating the sediment's surface was challenging where there was shell debris present: large shell fragments were moved aside until a more consolidated sediment was revealed in which the measurement was taken.

Redox values were converted to the hydrogen scale (temperature adjusted and the addition of a constant) following the protocol given by the Scottish Environment Protection Agency [37]. Following redox measurement the top 10 mm of sediment was removed by pushing the core upwards through a 10 mm ring and inserting a plastic ‘slicer’ through the sediment. Large shells or shell fragments were included in the sample where, as judged visually, they were mostly in the upper 10 mm. Once taken, sediment samples were stored on ice and then frozen (within 6 hours).

### Loss on ignition and particle size analysis

Frozen samples (see above) were freeze dried and their labile and refractory carbon content determined sequentially for approximately 5 g samples by weight loss on ignition (LOI) at 250°C and 500°C (8 hours each). It was assumed that negligible oxidation of non-organic carbon would occur at temperatures of <500°C [24].

Particle size analysis was done using a Coulter LS230 particle size analyser (Beckman-Coulter, Miami, USA). Prior to analysis, a 5–10 g aliquot of the remaining freeze-dried sediment was suspended in 15 ml distilled water to which 5 ml of an aqueous solution of 33 g sodium hexametaphosphate and 7 g sodium carbonate per litre (‘Calgon’) had been added. Each sample was thoroughly mixed using a vortex mixer and then left for approximately two hours. Immediately prior to analysis the samples were sonicated in a water bath for a minimum of 20 minutes and then passed through a sieve with a 1 mm screen to remove particles >1 mm which are too big for the Coulter counter method. This primary screening meant that large shell fragments were excluded from the particle size analysis.

Each sample was run three times by the Coulter LS230 to produce three estimates of the median particle size. The mean of these median values are reported here as the response variable particle size (PS). The median particle size was chosen as it is more robust, compared to the mean, to outliers and is a superior measure of central tendency where the particle size distribution is skewed [38].

### Loss on acid-digestion

The remainder of each sample, including any large shell fragments, was milled using a ball mill until reduced to a fine powder (no particles visible to the naked eye). Subsamples (approximately 250 mg) of the milled sediment were taken, weighed and then suspended in 50 ml of distilled water. Hydrochloric acid (1 M) was added until the solution remained acidic (∼pH 3). The suspension was agitated for 1 min, left for 5 min, the pH checked to ensure it was still acidic and then filtered under vacuum using pre-dried and weighed GF/F glass microfiber filter papers. Following filtration the filter papers were then dried until constant mass (∼4 hours at 80°C) and the weight loss determined and expressed as a carbonate percentage [39].

### Statistical analysis

Statistical analysis proceeded by plotting all data (box plots by site and bivariate associations), to assess heteroscedasticity, data clustering and colinearity [40]. Log transformations were applied to linearise any relationships as appropriate (distance was log_2_ transformed to facilitate interpretation). Redox was the response variable, with fixed effects distance, LOI250, LOI500, PS and carbonate content, and their interactions as potential predictors. Counter-intuitive interpretations of bivariate associations can occur where within and between Site associations are combined (i.e. ignoring Site). The potential for these was assessed visually and, for reasons of clarity, those with no observable pattern within Site are not shown.

The modelling of the relationship between redox and the predictors required a mixed model to account for the random factor ‘Site’ (see 0). Model development and selection in mixed models can be relatively complex, and iterative, and the guidance given in Zuur et al. [35], summarised below, was followed:

1. The data were firstly centred so that the model predicted intercept related to the predictors at their average values [41].
2. The beyond optimal (all fixed effects and interactions) model was initially fitted using linear regression and the residuals examined for homoscedasticity. If any trends were identified (versus Site or continuous predictors) a range of variance structures were tested and compared on the basis of their Akaike information criteria (AIC) score (where the lowest AIC is considered the optimal model). The goal was to identify and allow for differences in variance as a function of the predictors. Residuals from the model with the lowest AIC were reassessed to check that any heteroscedasticity had been incorporated into the model.
3. The next step was to identify the optimal random component (no random effect, random intercept and random intercept and slope). A variety of models were trialled based on a visual examination of Site-specific linear regressions to assess whether slopes/intercepts appeared to differ. Random components (intercepts and random slopes) were fitted (with the optimal variance structure, as above) and the optimal model chosen based on the lowest AIC.
4. The significance (or otherwise) of the fixed effects was then determined based on maximum likelihood (ML) parameter estimates. The full model was fitted and interactions/main effects sequentially tested using a likelihood ratio test. Non-significant terms were removed.
5. Model validation was then conducted based on an analysis of residual patterns. If the residuals were normally distributed standardised residuals were plotted against predictors, including random components. Patterns in the residuals resulted in a reassessment of the model and the adoption of non-linear models.
6. Where linear parameter estimates were not significant, or where residual patterns remained following data transformation, additive mixed models were trialled. The optimal variance structure and random components were determined as above but smoothed terms were added and their significance assessed (using the software default cross-validation setting). Non-significant smoothed terms were removed (and potentially retained as significant linear effects) and model validation conducted as above. Residuals were assessed as above. Final model parameters were determined using REML methods.

All statistical analyses were done using R [42]; linear mixed models (including generalised least square models) were constructed using R libraries ‘nlme’ [43] and ‘lme4’ [44], additive mixed modelling was done using the ‘mgcv’ library [45]. All spatial data were managed using ESRI ArcGIS™ (version 9.3)

## Results

### Site selection and description

**Figure 1:**
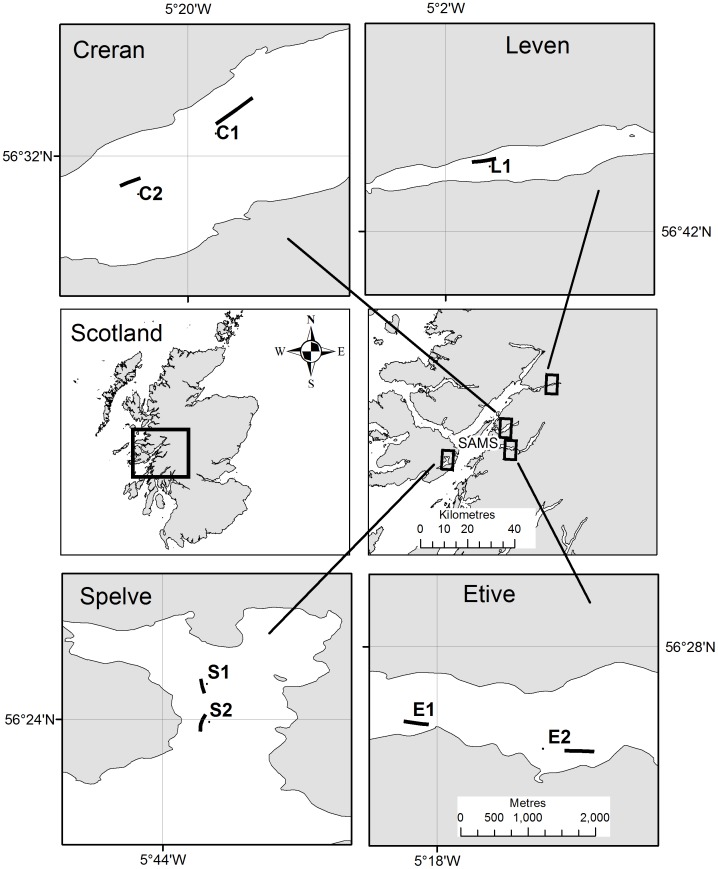
Sampling sites in Creran (C1 and C2), Etive (E1 and E2), Leven (L1) and Spelve (S1 and S2). The central map (left) shows Scotland, the area within the box is shown on the right. The sampled farm sites are shown in the large-scale maps, all 1∶50,000 (scale bar shown in Etive applies to all Loch maps). Latitude and longitude in degrees, minutes (WGS84) are shown on the larger scale maps. The location of the laboratory (‘SAMS’) is shown in the central map (right).

Farms located in four different lochs (Creran, Etive, Leven and Spelve) met the selection criteria. From each loch two long-line sites were randomly selected except in Loch Leven where there was only one suitable site (total = seven sites; Figure 1). The mussels were of a harvestable size at the Spelve and Leven sites (ca. 2 years old) whilst the Creran and Etive sites hosted a crop that was >2 years old and which had not been harvested for commercial reasons. Stocking density was approximately 400–600 individuals per metre. At low water the droppers at all sites extended through the entire water column stopping just above the seabed (in order to minimise attack from benthic predators). The sediment-water interface temperature ranged between 14.0 and 14.5°C. The Sites are briefly summarised in Table 1.

**Table 1:** Site summary and description.

Site	N	Depth (m)		Log_2_ distance (m)		Site description
		Mean	Sd	Mean	Sd	
C1	17	17	0.39	1.96	1.83	4 by 400 m mussel line array. Current speed: max 100 cm/s^A^
C2	23	16	0.51	2.62	1.22	4×200 m mussel line array, otherwise as above.
E1	17	34	1.37	2.74	2.46	4×200 m mussel line array. Current speed (200 cm/s^B^)
E2	23	25	3.25	3.36	1.72	3×200 m line array. River mouth 300 m to the south-west. Current speed: 100–200 cm/s^B^
L1	19	40	2.89	3.58	1.04	20×200 m closely spaced (3 m) array. River mouth at eastern end of site. Current speed: 100–200 cm/s^B^
S1	34	17	1.1	3.64	1.63	4×200 m mussel line array. Flat seabed. Flow: max 50 cm/s^C^
S2	26	23	0.99	2.83	1.71	As above.

N - sample size, Sd - standard deviation.

A - from unpublished fish-farm compliance monitoring data, fish-farm located approximately 1500 m south-west of the site C2.

B – as crudely estimated by monitoring maximum research vessel drift at the site during any of the surveys.

C – from unpublished acoustic Doppler current profiling, meter located equidistantly between S1 and S2.

### Technique evaluation and visual appearance of cores

Initially, collecting cores was hampered by the presence of shell hash (generally in close proximity to the mussel line). This problem was partly addressed by using longer core tubes (500 mm) which allowed greater penetration through any overlying mussel debris and into sediment that was sufficiently cohesive to seal the base of the core for a duration sufficient to remove it from the Craib corer. The presence of shell-debris at the surface also complicated inserting the redox probe (which had to be done by moving the shells aside) and both estimating and taking 10 mm sections (for subsequent sedimentary analysis). Sediment cores containing large-amounts of shell debris were usually dark brown or black in colour and frequently smelt of hydrogen sulphide.

### Sediment characterisation and bivariate associations

**Figure 2:**
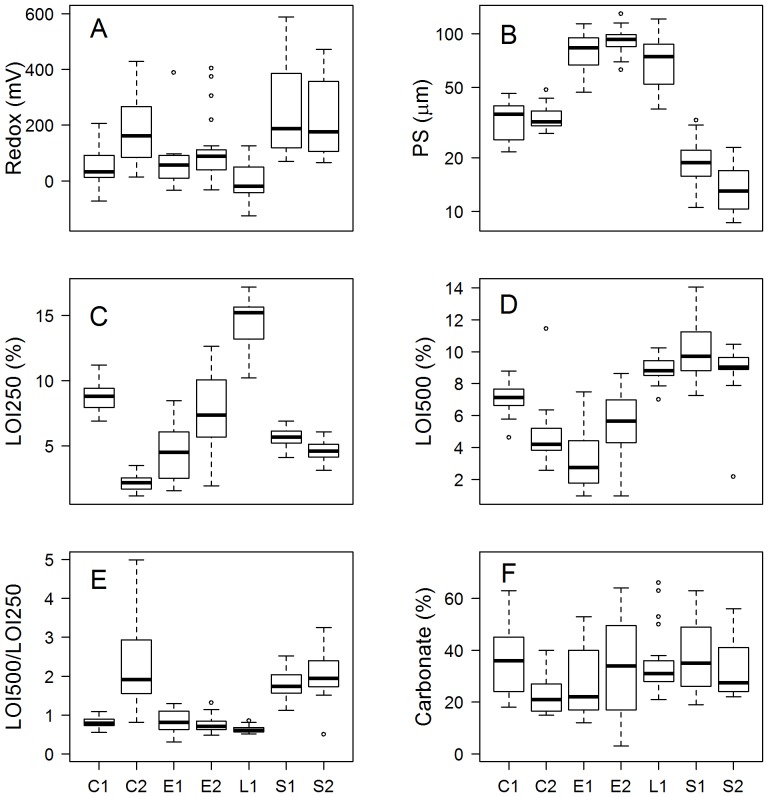
Sediment characterisation at different sites. A – particle size (log-scaled), B – loss on ignition (250°C), C – loss on ignition (500°C), D – ratio between LOI500 and LOI250 (one outlier, ratio = 5.8 is not shown for reasons of clarity), E – carbonate content (%) and F - redox. Site codes (X-axis): C - Creran, E - Etive, L - Leven, S - Spelve followed by site number (1 or 2). Sample sizes (per site) are shown in **Table 1**.

The sampled sediments ranged widely between sites with those from Etive and Leven being coarser (median PS>70 µm, **Table 2**) compared with Creran and Spelve sites which were much finer (median PS<35 µm; **Table 2**) corresponding to very-fine sands and silts respectively on the Wentworth scale [46]. Overall LOI (both 250 and 500) varied widely between sites (LOI250 range 1.7 to 17%, LOI500 range 1.0–14%, **Table 2**). The LOI at 500 and 250°C were approximately equal at Creran 1 and the Etive and Leven sites (ratio ∼1, **Figure 2**; **Table 2**), a trend not shared by Creran 2, where it ranged widely (ratio of 1 to 5 with an outlier of 5.8, not shown on **Figure 2** for reasons of clarity), or the Spelve sites where a ratio of approximately 2 was observed. Sediments from Leven demonstrated much higher LOI250 (mean 15%) compared to other sites (**Table 2**). Carbonate contents were highly variable (particularly at Etive 2) ranging between 3.0 and 66% (**Table 2**). Redox also varied widely across the sites ranging between −125 mV (Leven) and 588 mV (Spelve 1) (**Table 2**). Within sites the mean and range in redox was greatest at Spelve at approximately 240 mV with a standard deviation of approximately 150 mV (both sites, **Table 2**).

**Table 2:** Summary of sediment characteristics at the seven sites.

Site	N	Redox (mV)		LOI250 (%)		LOI500 (%)		PS (µm)		Carbonate (%)	
		Mean	Sd	Mean	Sd	Mean	Sd	Mean	Sd	Mean	Sd
C1	17	51	74	8.9	1.2	7.1	1.0	33	7.5	36	15
C2	23	185	124	2.2	0.66	4.7	1.8	34	5.5	22	6.5
E1	17	62	95	4.4	2.2	3.4	1.9	80	19	27	14
E2	23	106	117	7.6	2.9	5.6	1.9	92	15	34	20
L1	19	−3	73	14	2.1	8.9	0.80	73	24	36	13
S1	34	257	156	5.7	0.71	10	1.6	19	5.0	38	13
S2	26	236	145	4.6	0.76	8.9	1.5	14	4.4	33	10
Overall	159	147	152	6.5	3.8	7.3	2.8	46	32	33	14

N - sample size, Sd - standard deviation. Overall - across all sites. Site codes: C - Creran, E - Etive, L - Leven, S - Spelve followed by site number (1 or 2).

**Figure 3:**
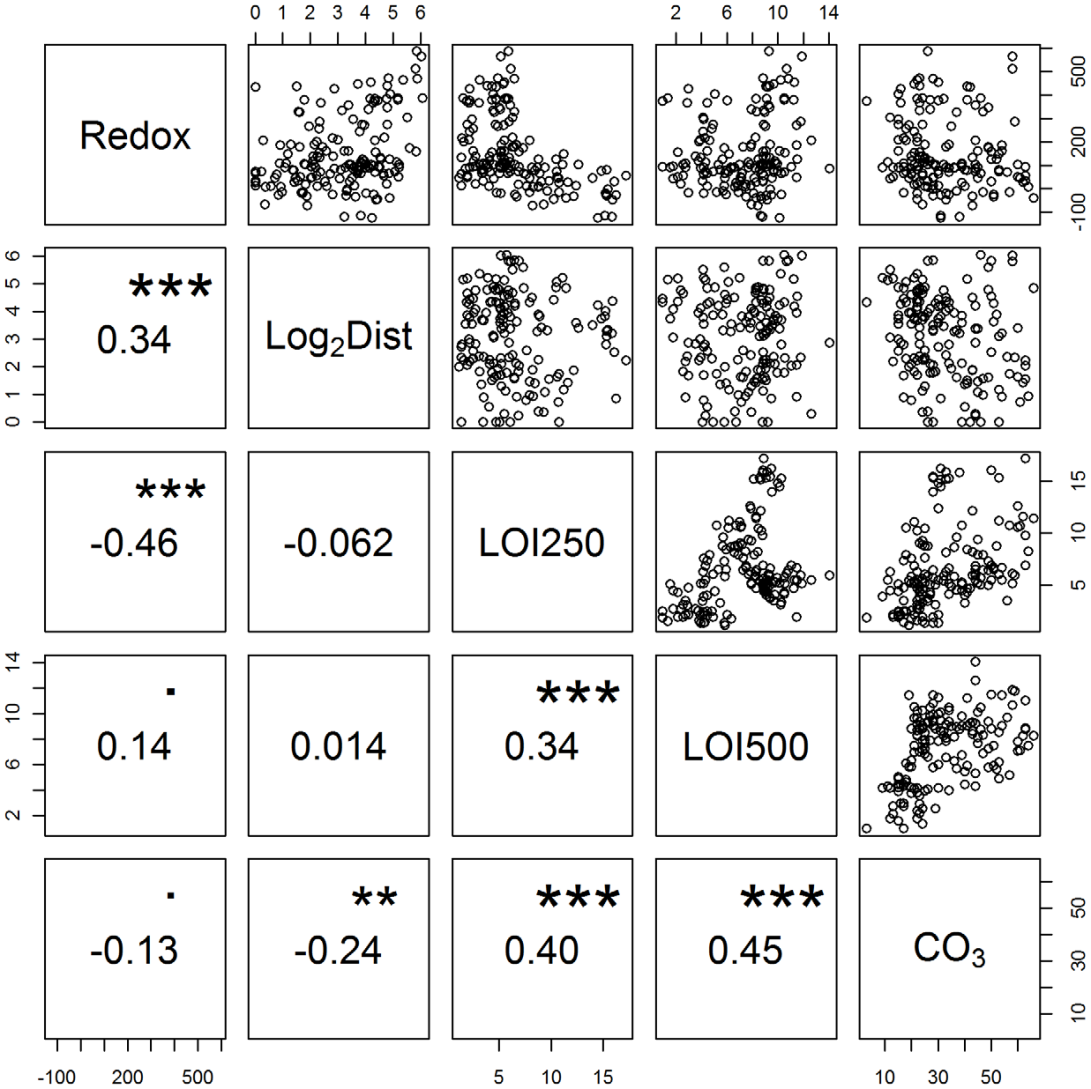
Bivariate correlations between response (Redox) and predictors across all sites. Log_2_Dist – log_2_ distance (m), LOI250/500 - loss on ignition at 250 and 500°C (%), CO_3_ – carbonate content (%). Correlations (font size scales with significance) and significances, are shown in the lower half of the plot. Significance of correlations key: *** - P<0.001, ** - P<0.01, * - P<0.05. N = 159.

In terms of simple bivariate associations, redox was positively associated with distance, (Log_2_Dist v Redox, **Figure 3**) and negatively associated with increasing labile organic content (Redox v LOI250, **Figure 3**). Carbonate content was positively associated with both LOI250 and LOI500 (**Figure 3**) and LOI250 and LOI500 were also positively correlated (**Figure 3**). Other correlations (not shown) indicated that sediments that were relatively coarse were associated with higher labile organic carbon but lower levels of refractory carbon. However, this apparent association occurred because both Spelve sites were characterised by a relatively low LOI250∶LOI500 ratio and fine sediments; these correlations simply reflected site differences and were not representative of trends within sites.

### Statistical modelling

The initial linear model resulted in residuals that varied considerably according to Site and Site was therefore used as a variance weighting factor in the analysis (different variances per Site were explicitly modelled). The optimal model also contained a random intercept for Site (**Table 3**) meaning that there were, overall, significant differences in redox between sites. The intercept standard deviation was relatively large (101 mV) compared with the residual (48 mV) and the weighting (indicating the relative standard deviation in redox per site) varied between 1.00 and 3.19 (**Table 3**). This showed that redox variability, both within and between Sites, is high even when the fixed components (discussed below) are incorporated into the model.

**Table 3:** Weighted GAMM results on the centred data (random and fixed effects).

Random effects		
	Intercept	Residual
Standard deviation	101	42.2

The modelled residual multiplier for each Site (weightings).						
Creran1	Creran2	Etive1	Etive2	Leven1	Spelve1	Spelve2
1.00	2.72	1.87	1.97	1.72	3.02	3.19

Fixed effects			
Factor	Estimate	Std. error	P
Intercept	118	41.5	**0.00472**
500	−14.9	5.78	**0.0109**
250	5.48	5.79	0.345
PS	−28.1	30.0	0.351
500:250	−4.20	2.03	**0.0401**
500:PS	−0.649	8.39	0.938
250:PS	−4.03	6.00	0.502
500:250:PS	6.97	2.21	**0.00195**
Smoothed component			
	edf	F	P
s(Log_2_Dist)	2.95	9.97	**5.77×10^−6^**

Site was used as both a weighting factor and a random intercept in the model. Key: estimate - regression coefficient estimate, 250 - LO250, 500 - LOI500, PS - particle size (log transformed), (s)Log_2_Dist - distance from line (m) (log_2_ transformed and smoothed), edf - estimated degrees of freedom (degree of smoothing), F - F ratio, P - probability under null hypothesis of no-effect. Significant effects (P<0.05) are highlighted in bold.

**Figure 4:**
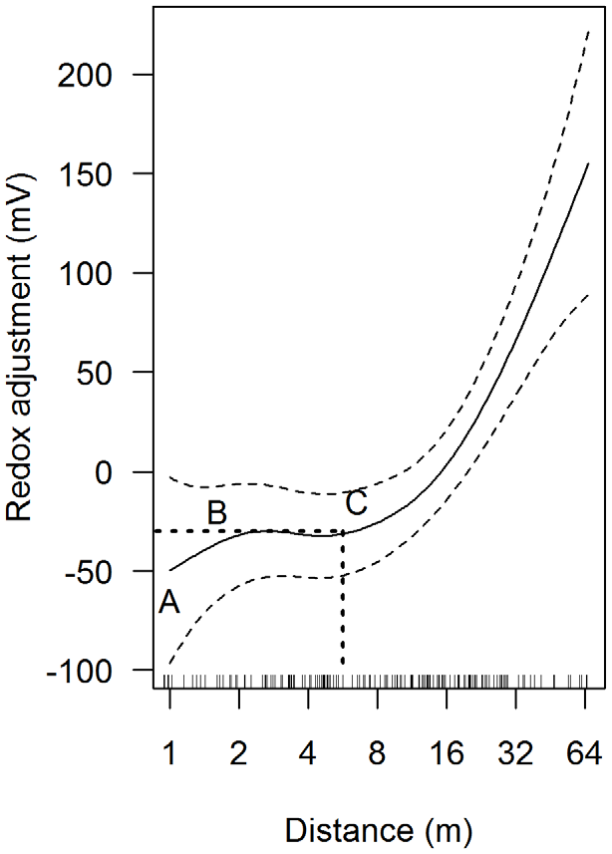
GAMM modelled relationship between Log_2_Dist (m) and redox. The predicted redox, at any distance, should be read off the Y axis and then added to the model intercept estimate given in Table 2 (e.g. at a distance of ∼6 m the model predicts a redox of the model intercept minus 30 = 88 mV). The rug (jittered) on the X axis indicates distances where sampling occurred (N = 159). The regions A to B, B to C and C onwards are explained in the text.

Whilst the optimal linear model was a reasonable fit, the resultant residuals demonstrated non-linear trends when plotted against Log_2_Dist. This was confirmed by fitting a generalised additive mixed model (GAMM with the identity link function) which indicated a relatively simple, and highly significant, non-linear relationship between redox and Log_2_Dist. The optimal model (smoothing estimated degrees of freedom = 2.95, P<0.0001; **Table 3**) is shown in **Figure 4**.

### Details of the optimal model

The relationship between line-proximity on redox can be divided into three areas: from 1 m to 2 m (A to B), from 2 m to 7 m (B to C) and greater than 7 m (C onwards) (**Figure 4**). Under average conditions (of LOI and PS; as shown in **Table 2**), at 1 m distance from the mussel line, modelled redox was reduced by 50 mV (95% confidence interval 10 and 100 mV) compared to the average redox value over the sampling domain. Over the mussel-line distance 2–7 m this reduction was approximately 30 mV (95% confidence interval 10 and 55 mV) whilst at distances >7 m the effect of mussel line proximity diminished rapidly (**Figure 4**). Therefore, given that the intercept was >100 mV (Table 3) the model predicted that, under average conditions at the average site, negative redox values are unlikely even adjacent to mussel farms.

The main factors (in addition to Dist and Site) that influenced redox were organic content (LOI250 and LOI500) and particle size (significant coefficients shown in **Table 3**). The effect of these factors effectively moves the curve shown in **Figure 4** up or down (without changing the shape of the distance-redox relationship). Of the measured covariables LOI500 had the greatest independent effect and was negatively linked to redox with a 1% increase resulting in a predicted 15 mV reduction in redox. However, this effect was complicated by an interaction with LOI250 (**Table 3**) and with both LOI250 and particle size (**Table 3**): setting both LOI250 and LOI500 at 2.5% above average, to LOI of approximately 9 and 10% respectively (levels within the range recorded here) and equating to an organically enriched sediment, resulted in a predicted redox reduction of 50 mV (irrespective of distance such that the curve shown in **Figure 4** is dropped by 50 mV). Under these circumstances the model predicts that negative redox values can be expected adjacent to mussel lines (1 m, A in **Figure 4**) and are plausible (approximately within the 95% confidence intervals) up to 7 m from the edge of the mussel farm (B–C in **Figure 4**). Terms including carbonate did not make a significant contribution to the model and were not included.

## Discussion

Quantifying the benthic changes occurring around mussels farms was challenging from both a practical and statistical modelling perspective. However, significant reductions in redox were found in close proximity to farms and the functional relationships between farm distance and benthic parameters were determined. These findings have a number of implications for the monitoring and management of the expanding mussel culture industry which are discussed below.

### Methodological issues

The research reported here is one of the most comprehensive cross-sectional studies which has focussed on the lateral extent and nature of sedimentary changes associated with mussel farms (see also [47]). However, any observational programme is limited through the logistics of site access and sampling time and cost. In the current case, in order to collect numerous spatially independent samples, within a limited budget, a remote sampling method was used (as opposed to the diver method commonly used in other related research e.g. [48]). Sampling using a standard Craib corer around mussel lines was challenging particularly adjacent to the line where shell debris was more commonly observed in the cores. Whilst longer core tubes and greater ballast on the corer enhanced coring success, the presence of shell debris (which included whole shells) will always make estimating the sediment's surface (and hence 10 mm depth) difficult.

Accounting for the natural variability in response variable between sites is a major challenge in designing observational sampling programmes that are capable of identifying and describing the spatial extent of benthic impacts, particularly where those impacts are relatively modest. In the current case this was achieved through taking sufficient samples and focussing on the seaward side of the mussel lines, combined with the modelling approach which allowed for random and non-linear effects. In a cross-sectional study all samples would, ideally, be collected instantaneously but larger sampling programmes necessarily extend over time. The degree of seasonal change over the period July–September recorded here, at least in terms of water temperature, is relatively small (<1°C) and any temporal differences would be accounted for in the random effect ‘Site’ because different sites were visited sequentially. Temporal changes were, therefore, discounted in the reported models.

In field sampling programmes there are numerous covariables which could, logically, be measured and included in the analysis. However, in any model the parameter estimates become increasingly less reliable as the ratio between numbers of observations and number of parameters decreases particularly where the response shows high variance. In the current case sedimentary organic content was included because organic enrichment, particularly of labile material (LOI250) has been considered a potential driver of change (see Introduction) whilst particle size was cost-effectively measured and is closely associated with current speed (considered a likely moderator of impact, see Introduction). The proportion carbonate indicated the degree to which shell debris dominated the sediment, potentially enhancing the entrapment of organic material, and was also included.

### Changes in sediments around mussel farms

The results presented here are the first to demonstrate the functional relationship between mussel-line distance and redox, and the associated effect of particle size and organic enrichment. In this way it addresses what has been identified as a major gap in our understanding of mussel farm – environment interactions [23]. Despite the inherent variability in redox (between and within sites) a clear pattern emerged of an expected redox reduction of 50 mV (compared to the mean at any given site) at the immediate edge of the farm, moderation of this impact (expected redox reduction of 30 mV) from 2–7 m and an increase thereafter. The model suggests that where organic enrichment occurs (LOI250 and 500 both ∼10%) redox levels of less than zero are likely adjacent to the line and plausible up to 7 m from the line.

The predicted 10 to 100 mV reduction in redox, at the farm's periphery, is similar to the zero to 120 mV difference reported by Cranford *et al* [49] from eleven embayments in Prince Edward Island, Canada, but slightly less than the 50–200 mV difference found by Hargrave et al [29] who focussed on Tracadie Bay (Prince Edward Island again, values based on comparing 95% confidence intervals determined from lease and non-lease (no *Zostera*) stations) and the >200 mV reduction in redox recorded by both Mattson and Lindéen [50] and Chamberlain et al [31] (both based on a single transect with no replication). The reduction in redox is likely to be attributable to mussel-farm related biodeposit flux to the seabed. However, the observed pattern (of non-linearly increasing redox with distance) may reflect both the non-linear dispersion of biodeposits around the mussel line and/or the lateral movement of the mussel lines both of which may blur the definition of the farm boundary.

The significance to the benthos of the reductions in redox of the scale reported here will be dependent on the receiving environment. Where background sedimentary redox is naturally high (>∼200 mV) a reduction of up to 100 mV within the boundaries of the farm may only have a minimal impact [26] and the addition of organic matter is likely to increase macrobenthic biomass without reducing biodiversity, as has been reported around some mussel farms (e.g. [51] and [32] respectively). However, in other environments the lowering of redox by 10–100 mV may have more negative consequences such as a reduction in benthic infaunal biodiversity such has been observed around mussel farms in a variety of locations including South Africa [52], Spain [53], New Zealand [54], Ireland [31] and Italy [55].

In the current case, there was a negative correlation between sediment carbonate content (a proxy for shell content) v. distance and between labile carbon v. redox and a positive association between both labile and refractory organic content and carbonate. These bivariate associations and the absence of carbonate in the statistical model (suggesting that its effects are accounted for by other factors) are commensurate with the hypothesis that shell debris traps organic material [23], as has also been reported for maerl in relation to fish-farm-derived organic material [56], mediating the reduction in sedimentary redox. However, further research is required to definitively separate the effects of shell-hash and organic matter accumulation given that hash was more prevalent adjacent to the mussel lines.

The sampling reported here occurred during the summer when water temperatures were at a maximum (ca 14.5°C) and when phytoplankton food is relatively abundant in the Lynn of Lorne (which connects to the lochs reported here) [57]. In these relatively warm conditions impacts could be expected to be at their greatest because of elevated rates of microbial decomposition, minimal oxygen solubility [18] in a situation of on-going biodeposition from feeding mussels. Whilst the results reported here do not demonstrate how sedimentary redox (and covariables) vary over time they are likely to represent an assessment of the lateral extent of impacts at approximately their yearly maxima. However, the modification of sediments within the farm boundary is likely to be greater than those occurring at the farm periphery (as reported here) because of the enhanced baffling of water currents within the farm and concomitantly greater biodeposit accumulation [58]–[60].

### Recommendations to regulators

This research supports the hypothesis that changes in sediment biogeochemistry will occur around mussel farms and that these may be associated with the presence of mussel shell debris. For farms on the west-coast of Scotland these results indicate that impacted zones around mussel farms (negative redox values) will extend less than 7 m, even where the sediment is moderately organically enriched. Given the spacing between mussel lines (generally 5–15 m in Scotland), these results indicate that unmodified sediment between lines is unlikely and, therefore, a conservative estimate of the footprint size of a mussel farm could include the area under the farm plus a 7 m boundary. Where farms are located over sediments with background redox values of <100 mV, the farm footprint may manifest itself as a region of reduced biodiversity associated with anoxic sediments and high sulphide concentrations. Conversely, where sediments are naturally highly oxic, the farm footprint is more likely to be defined by increased biodiversity and/or benthic productivity.

The mechanism by which mussel hash influences redox potential may extend beyond the entrapment of organic matter. Separating the independent effects of shell debris from any role it has in trapping organic material is a major challenge particularly given that there is little known about the longevity of shell hash or its impacts once buried by sediment. However, these results suggest that polyculture operations (combining fin-fish and mussels in close spatial proximity or where sites are interchanged) may have more serious consequences for benthic communities at the scale of the mussel farm via enhanced entrapment of fish-farm derived organic matter in shell-hash. Husbandry practices that result in the disposal of mussels onto the seabed should be avoided where the objective is to minimise impacts.

The results presented here only apply to sedimentary environments. The impacts of mussel farming on hard-substratum benthic assemblages have not been investigated. Addressing this knowledge gap would enable a better prediction of the benthic impacts of an expanding mussel growing sector.
